# Influence of Enzymatic Catalysis and Reduced Application Time on Enamel Chemical and Structural Changes Induced by In-Office Bleaching

**DOI:** 10.3390/jfb17100531

**Published:** 2026-10-20

**Authors:** Lara Maria Bueno Esteves, Cristina de Mattos Pimenta Vidal, Marcelle Danelon, Carlos Alberto de Souza Costa, Ariene Leme-Kraus, Erika Michelle dos Santos Araujo, Senthiuguru Kulanthaivel, Karen Milaré Seicento Aidar, André Luiz Fraga Briso

**Affiliations:** 1 Department of Operative Dentistry and Endodontics, Araçatuba School of Dentistry, São Paulo State University (UNESP), Araçatuba 16015-050, SP, Brazil; lm.esteves@unesp.br (L.M.B.E.); karen.milare@unesp.br (K.M.S.A.); 2 Department of Operative Dentistry, College of Dentistry, The University of Iowa, Iowa City, IA 52242, USA; cristina-vidal@uiowa.edu (C.d.M.P.V.); senthilguru-kulanthaivel@uiowa.edu (S.K.); 3 Department of Operative Dentistry, Periodontology and Pediatric Dentistry, Faculty of Medicine and University Hospital Carl Gustav Carus, TUD Dresden University of Technology, Fetscherstraße 74, 01309 Dresden, Germany; marcelle.danelon@ukdd.de; 4 Department of Physiology and Pathology, Araraquara School of Dentistry, São Paulo State University (UNESP), Araraquara 14801-903, SP, Brazil; cas.costa@unesp.br; 5 College of Dental Medicine-Arizona, Midwestern University, Glendale, AZ 85308, USA; edossa@midwestern.edu

**Keywords:** dental biomaterials, esthetic dentistry, tooth bleaching, hydrogen peroxide, ATR-FTIR, EDS, ROS, enamel

## Abstract

**Objectives:** This study evaluated a bleaching strategy combining horseradish peroxidase (HRP) and a polymeric scaffold with 35% hydrogen peroxide, compared with a conventional in-office bleaching protocol. **Methods:** The study was conducted in two experimental phases. In Phase I, enamel from five sound human premolars was pooled, homogenized, and divided into technical aliquots for ATR-FTIR analysis and quantification of reactive oxygen species (ROS). In Phase II, 24 human premolars were allocated to four experimental groups (*n* = 6 per group) to evaluate surface microhardness, post-treatment surface roughness, and elemental composition. Four protocols were investigated: NC (negative control), G45 (35% H_2_O_2_ for 45 min), G15 (35% H_2_O_2_ for 15 min), and G15SP (polymeric scaffold + HRP for 15 min). **Results:** G15SP exhibited lower post-treatment surface roughness and greater preservation of enamel microhardness compared with the conventional G45 protocol. Exploratory ATR-FTIR analysis showed relatively stable PO_4_^3−^/CO_3_^2−^ ratios in NC, minor variations in G15SP, and the greatest treatment-associated variations in G45. Descriptive ROS analysis showed the highest initial ROS generation in G15SP. EDS analysis showed a lower Ca/P weight ratio in G45, whereas G15SP maintained values closer to those observed in the untreated control. **Conclusions:** Enzymatic catalysis combined with a polymeric scaffold was associated with greater preservation of enamel elemental composition, surface characteristics, and mechanical properties compared with the conventional 45-min bleaching protocol. **Clinical significance:** Modulation of hydrogen peroxide reactivity through enzymatic catalysis and a polymeric scaffold may represent a promising strategy to reduce the structural impact of in-office bleaching while shortening peroxide exposure time. Its clinical bleaching efficacy should be confirmed in studies specifically designed to evaluate color change.

## 1. Introduction

In-office tooth bleaching is widely used to meet the growing demand for fast and effective esthetic treatments [1]. This procedure commonly employs hydrogen peroxide (H_2_O_2_) as the bleaching agent, generally at concentrations ranging from 25% to 40% [2]. Although higher peroxide concentrations and prolonged application times may enhance bleaching efficacy [3,4,5], these aggressive protocols have also been associated with adverse effects, including tooth sensitivity [6,7], gingival irritation [7], and enamel demineralization [8]. Since dental enamel acts as a protective barrier against chemical, physical, and mechanical challenges [9,10], preserving its structural integrity is essential for maintaining tooth homeostasis [11].

Traditional bleaching protocols have been reported to induce microstructural changes in enamel, influenced by factors such as formulation pH [12], exposure time [13], and bleaching agent concentration [14]. These alterations may involve the loss of calcium and phosphate ions [15], which can compromise enamel integrity and reduce surface and subsurface microhardness [16,17]. Furthermore, bleaching procedures may promote the formation of microporosities and subtle changes in enamel surface roughness, potentially affecting the surface characteristics of dental enamel [18].

A promising strategy to reduce clinical time and minimize the deleterious effects of bleaching treatment involves accelerating the decomposition of H_2_O_2_ into more reactive free radicals, such as hydroxyl and perhydroxyl radicals [19]. In this context, the enzyme horseradish peroxidase (HRP) has been investigated as a catalytic agent capable of accelerating hydrogen peroxide decomposition, enhancing the generation of reactive oxygen species responsible for the bleaching reaction, and reducing residual free H_2_O_2_, thereby potentially enabling effective whitening with shorter application times [20,21].

Polymeric scaffolds have also been proposed as adjunctive biomaterials that may act as protective barriers capable of modulating the diffusion of H_2_O_2_ through dental tissues. These structures can also function as support matrices for enzymatic systems, allowing the immobilization or stabilization of catalytic agents within bleaching formulations [20,21]. Thus, when incorporated into bleaching protocols containing peroxidase, polymeric scaffolds may indirectly contribute to the catalytic process while limiting the trans-amelodentinal diffusion of residual peroxide toward the pulp complex. Ortecho-Zuta et al. (2022) [20] proposed a protocol combining enzymatic catalysts and polymeric scaffolds in a 15-min treatment, reporting sustained esthetic outcomes and a significant reduction in the amount of residual H_2_O_2_ reaching the pulp complex, thereby mitigating the biological impact of bleaching agents on pulp cells [21,22,23].

Previous studies from our research group [24,25,26] have demonstrated that the association of enzymatic catalysts with polymeric scaffolds produces bleaching outcomes comparable to conventional in-office bleaching protocols, even with a 1/3 reduction in the contact time of the bleaching gel with the dental structure [26,27,28]. These findings support the potential of this strategy to achieve clinically relevant color change while reducing treatment time. However, despite the promising whitening efficacy previously reported, the effects of this catalytic approach on the dental substrate remain poorly understood. Therefore, further investigations are needed to determine whether the reduction in clinical time and the use of enzymatic catalysts associated with optical activation may influence the chemical and structural properties of dental enamel.

Therefore, the aim of this study was to evaluate the effects of an enzymatic catalytic strategy consisting of horseradish peroxidase (HRP) associated with a polymeric scaffold combined with a 35% H_2_O_2_ bleaching gel, compared with a conventional in-office bleaching protocol. The specific objectives were to: (1) evaluate enamel topography and microstructure through imaging, surface roughness, and microhardness analyses; (2) assess changes in enamel chemical structure before and after treatment; (3) compare the generation of reactive oxygen species (ROS) during the bleaching procedures; and (4) analyze the elemental composition of enamel following treatment. The tested hypotheses were that, compared with the conventional bleaching protocol, the catalytic strategy would: (1) influence enamel surface roughness and microhardness; (2) reduce chemical alterations in enamel; (3) minimize changes in enamel elemental composition; and (4) increase ROS generation.

## 2. Methodology

Ethical review and approval were waived by the Institutional Review Board of the University of Iowa because the study used extracted human teeth that had been removed for clinical indications unrelated to the purposes of this research. No identifiable patient information was collected or used, and patient anonymity was fully preserved throughout the study.

### 2.1. Study Design

The study was conducted in two experimental phases designed to evaluate the chemical and structural effects of different bleaching protocols on dental enamel.

**Phase I** evaluated the chemical reactions involved in the bleaching process using enamel powder. ATR-FTIR analysis and quantification of reactive oxygen species (ROS) were performed to investigate chemical changes in enamel and reactive species generation during the bleaching procedures.

**Phase II** evaluated the effects of the bleaching protocols on intact enamel surfaces using human tooth specimens. Surface microhardness, roughness, energy-dispersive spectroscopy (EDS), and 3D laser confocal microscopy were used to assess structural and compositional changes after treatment.

The use of enamel powder was adopted to increase the sensitivity and reproducibility of the chemical analyses performed in Phase I. Intact enamel surfaces present intrinsic morphological variability, including differences in prism orientation, surface curvature, and local mineral density, which may introduce analytical variability and interfere with spectroscopic and chemical measurements. By converting enamel fragments into a standardized powder with a controlled particle size, the exposed surface area became more homogeneous, allowing a more uniform interaction between the bleaching agent and the mineral substrate. This approach minimized sample-to-sample variability and enhanced the sensitivity for detecting chemical alterations and reactive oxygen species generated during the bleaching process. Although this model provides a larger contact area between the substrate and the bleaching agent than intact enamel surfaces, it was intentionally employed in Phase I to facilitate the detection of chemical changes under standardized experimental conditions. In contrast, Phase II was performed using intact enamel specimens to evaluate the structural and morphological effects of the treatments under conditions that more closely resemble the clinical scenario.

The experimental bleaching protocol and specimen preparation procedures were based on methodologies previously established and validated by our research group [24,25,26,27]. However, the novelty of the present study lies in the comprehensive mechanistic evaluation of enzymatically catalyzed bleaching through the integration of reactive oxygen species generation, ATR-FTIR, energy-dispersive X-ray spectroscopy, surface microhardness, surface roughness, and three-dimensional confocal microscopy. The combination of these complementary analytical techniques enabled a broader understanding of the relationship between oxidative activity, mineral composition, and enamel structural integrity than has been reported in our previous studies.

The experimental design considered two main factors:

(1) Bleaching Protocol (four levels):
  1. Negative Control (NC)—no bleaching treatment;
  2. Conventional Protocol (G45)—application of bleaching gel with 35% H_2_O_2_ directly to the enamel for 45 min;
  3. Short Protocol (G15)—application of bleaching gel with 35% H_2_O_2_ directly to the enamel for 15 min;
  4. G15SP—enamel covered with a nanofiber scaffold followed by application of bleaching gel with 35% H_2_O_2_ containing horseradish peroxidase (HRP) for 15 min.
(2) Evaluation times (four levels):

Baseline (Initial); after the first bleaching session (S1); after the second session (S2); and after the third session (S3).

However, the evaluation times varied according to the type of analysis performed. ATR-FTIR analyses were conducted at all points (Initial, S1, S2, and S3) to monitor changes in the chemical structure of enamel throughout the bleaching process. The generation of reactive oxygen species (ROS) was assessed at baseline and after the final bleaching session (Initial and S3). Surface roughness and microhardness were evaluated at baseline and after completion of the bleaching protocol (Initial and S3). Elemental composition by Energy Dispersive Spectroscopy (EDS) was assessed only after the final bleaching session (S3).

The following response variables were evaluated:

1. Chemical structure of enamel (ATR-FTIR);
2. Generation of reactive oxygen species during bleaching;
3. Surface topography and morphology (3D confocal microscopy);
4. Surface roughness;
5. Surface microhardness;
6. Elemental composition of enamel (EDS).

### 2.2. Phase I—Enamel Powder

#### 2.2.1. Sample Preparation

Five sound human premolars obtained from the tooth bank of the University of Iowa (Iowa City, USA) were used in Phase I of this study.

After extraction, the teeth were cleaned in an ultrasonic bath with distilled water for 2 min to remove biological debris and blood residues. Before specimen preparation, all teeth were carefully examined under magnification using a dental loupe to assess their structural integrity. The inclusion criteria comprised recently extracted teeth without caries, restorations, structural defects, coronal or root fractures, extensive cracks, severe tooth wear, enamel hypomineralization, or root resorption. Teeth presenting enamel cracks, fractures, or other visible structural defects were excluded and replaced to ensure sample standardization.

The crowns were sectioned mesiodistally using a precision diamond saw (Isomet 1000, Buehler Ltd., Lake Bluff, Illinois, USA) under continuous water irrigation to separate the buccal and lingual surfaces. Internal dentin and residual pulp tissue were carefully removed using diamond burs under magnification, and the external cementum was eliminated to isolate the enamel fragments. All prepared enamel fragments were examined under a stereomicroscope to confirm the absence of dentin, cementum, or other non-enamel tissues. Fragments containing residual non-enamel tissues were excluded and replaced.

The enamel fragments obtained from all five teeth were then combined to form a single pooled enamel preparation. The pooled enamel was briefly dried in acetone for 5 s and subsequently immersed in liquid nitrogen for 5 min to facilitate cryogenic grinding. The pooled enamel was ground in a cryogenic ball mill (CryoMill, Retsch GmbH, Haan, Germany) at 30 Hz for 1 min to obtain enamel powder. The resulting powder was sieved through a #400 mesh to standardize particle size distribution and thoroughly homogenized.

Pooling was intentionally performed to minimize inter-specimen variability in enamel mineral composition and to ensure that all experimental groups originated from the same standardized enamel substrate. This approach was selected because Phase I was designed as an exploratory mechanistic experiment focused on treatment-related chemical and oxidative effects rather than on inter-individual biological variability. Thus, using a common pooled substrate allowed potential differences in the initial mineral composition of individual teeth to be controlled across experimental conditions.

The homogenized enamel powder was subsequently divided into twenty 1-g aliquots, with five aliquots allocated to each experimental condition: NC, G45, G15, and G15SP. Therefore, the five aliquots within each treatment group were technical subsamples derived from the same pooled enamel preparation and were not considered independent biological replicates. The use of enamel powder increased the exposed surface area and promoted greater homogeneity during the spectroscopic and chemical analyses.

The number of aliquots per group was based on a previous study with a comparable experimental design [27]. Whereas the previous investigation used three aliquots per experimental condition, five technical aliquots per condition were used in the present study to increase the number of repeated measurements within the standardized pooled substrate and improve the reproducibility of the experimental observations.

#### 2.2.2. Fourier-Transform Infrared Spectroscopy with Attenuated Total Reflectance (FTIR-ATR) Analysis

A spectrophotometer equipped with a single-reflection ATR accessory with a diamond crystal (PIKE MIRacle ATR, Madison, WI, USA) coupled to an FTIR spectrometer (Vertex 80, Bruker, Billerica, MA, USA) was used for spectral acquisition.

Five aliquots of 1 g of enamel powder [27] were used for the FTIR analysis. Each aliquot was carefully deposited on the ATR crystal surface and gently pressed to ensure uniform contact between the sample and the crystal. For each sample, spectra were collected in the range of 450–4000 cm^−1^, with a spectral resolution of 4 cm^−1^. Each spectrum corresponded to the average of 64 consecutive scans.

Spectra were obtained at baseline and after the experimental sessions. The acquired spectra were processed to reduce potential water interference and were subsequently normalized and baseline-corrected using OMNIC software (v7.0, Thermo Scientific, Madison, WI, USA).

The mineral composition of enamel was analyzed based on the absorbance intensity and peak area of the carbonate band (871 cm^−1^) and phosphate band (1019 cm^−1^). Additionally, the ratio between the areas of the phosphate and carbonate peaks was calculated to assess potential alterations in enamel mineral structure following the experimental treatments [28].

#### 2.2.3. Scaffold Fabrication

Polymeric scaffolds (ScPs) were fabricated by electrospinning using a 12.5% (*w*/*v*) polycaprolactone (PCL; Sigma-Aldrich Corp., St. Louis, MO, USA) solution prepared in a chloroform/dimethylformamide solvent system (80:20 *v*/*v*) (Labsynth Produtos para Laboratório, São Paulo, SP, Brazil). The polymer solution was transferred to a sterile 5 mL syringe (Luer-Lock BD, Curitiba, PR, Brazil) fitted with an 18-gauge stainless steel hypodermic needle (Bunzl Saúde/Labor Import, São Paulo, SP, Brazil).

The syringe was connected to a programmable syringe pump (KDScientific, Holliston, MA, USA) set at a flow rate of 0.5 mL/h. Electrospinning was performed under an applied voltage of 12 kV, with the needle tip positioned 15 cm from the collector. To produce a randomly oriented nanofiber mesh, a flat aluminum collector was placed perpendicular to the needle. The electrospinning process was maintained for 45 min, after which the resulting nanofiber scaffold was collected and stored for subsequent use.

The obtained mesh was disinfected by immersion in 50% ethanol for 10 min, followed by immersion in 70% ethanol for 20 min. This disinfection cycle was repeated three times. Subsequently, the scaffold was immersed in phosphate-buffered saline (PBS) for 10 min and allowed to dry at room temperature for 48 h before use [20].

#### 2.2.4. Bleaching Treatment

The prepared enamel powder was distributed into individual 1.5 mL microcentrifuge tubes (Eppendorf^®^, Hamburg, Germany) for the bleaching procedures. The bleaching agent used was a 35% hydrogen peroxide gel (Whiteness HP, FGM Produtos Odontológicos, Joinville, SC, Brazil), prepared according to the manufacturer’s instructions by mixing three drops of hydrogen peroxide with one drop of thickener.

A standardized volume of 0.06 mL of bleaching gel was applied using a positive displacement micropipette suitable for viscous liquids (Microman E, Gilson Inc., Middleton, WI, USA). The exposure time varied according to the experimental group.

The treatments were distributed into the following groups:

1. Negative Control (NC): The enamel powder was kept without bleaching treatment.
2. G45 (Conventional Protocol): The enamel powder was initially positioned at the bottom of the microcentrifuge tube. Subsequently, the standardized volume of bleaching gel was dispensed into the tube. The powder and gel were manually homogenized using a spatula until a uniform mixture was obtained and no visible powder residue remained. The mixture remained in contact with the gel for 45 min.
3. G15 (Short Protocol): The enamel powder was initially positioned at the bottom of the microcentrifuge tube. Subsequently, the standardized volume of bleaching gel was dispensed into the tube. The powder and gel were manually homogenized using a spatula until a uniform mixture was obtained and no visible powder residue remained. The mixture remained in contact with the gel for 15 min.
4. G15SP (Scaffold + Gel with Peroxidase): The enamel powder was placed at the bottom of the microcentrifuge tube, similar to the other experimental groups. However, in this group the bleaching gel was not applied directly onto the powder. Instead, the enamel powder was first covered with the electrospun scaffold, forming an intermediate layer between the powder and the bleaching agent. Subsequently, the bleaching gel containing 35% hydrogen peroxide was combined with 10 mg of horseradish peroxidase (HRP; Sigma-Aldrich, St. Louis, MO, USA) and deposited over the scaffold surface. This configuration allowed the bleaching agent to interact with the enamel substrate through the scaffold layer during the 15-min exposure period.

The 45-min application protocol (G45) followed the manufacturer’s recommendation for conventional in-office bleaching. The 15-min application protocol (G15) was included as an experimental control to evaluate the effect of reducing the application time without the addition of catalysts. This protocol was designed to match the application time used in the catalyzed group, enabling the isolated effect of the bleaching gel to be assessed under the same experimental conditions.

#### 2.2.5. Washing and Preparation of the Powder

After each bleaching session, the microcentrifuge tubes containing the samples underwent a standardized washing protocol to remove residual bleaching gel. An 800 µL amount of distilled water was added to each tube, followed by vortex mixing for 60 s to ensure suspension homogenization. Subsequently, the samples were centrifuged at 5000× *g* for 10 min at 23 °C. The supernatant obtained from the first centrifugation was carefully collected for the quantification of residual ROS. Subsequently, four additional washing cycles were performed by replacing the supernatant with fresh distilled water, followed by vortex agitation and centrifugation under the same conditions, to ensure the effective removal of bleaching gel residues.

After the final centrifugation step, the samples were prepared for lyophilization. The microcentrifuge tubes were sealed with parafilm perforated using a 30-gauge hypodermic needle to allow controlled moisture evaporation. The samples were then frozen at −80 °C for 24 h and lyophilized for 2 h to remove residual water.

This procedure was repeated after each bleaching session to ensure standardization of the experimental conditions and preservation of the samples for subsequent analyses.

#### 2.2.6. Quantification of Reactive Oxygen Species (ROS)

The generation of reactive oxygen species (ROS) produced during the bleaching procedures was quantified using the fluorescent probe carboxy-H_2_DCFDA (Invitrogen, San Francisco, CA, USA). Analyses were performed in sterile black 96-well microplates (KASVI Imp., São José dos Pinhais, PR, Brazil) to minimize background fluorescence.

Five aliquots of the freshly prepared bleaching gel and five aliquots of the supernatant obtained from each experimental treatment were dispensed into the wells of the microplates. Subsequently, the fluorescent probe carboxy-H_2_DCFDA was added to each well at a final concentration of 1 mM, maintaining a 1:1 ratio relative to the sample volume.

Fluorescence intensity was measured using a multimode microplate reader (Synergy H1, BioTek Instruments, Winooski, VT, USA) with excitation at 492 nm and emission at 527 nm. The obtained fluorescence values were used to compare the relative ROS production among the bleaching protocols.

### 2.3. Phase II—Human Tooth Samples

#### 2.3.1. Sample Preparation

Twenty-four sound human premolars extracted from individuals aged 18–30 years were used in Phase II and allocated to four experimental groups (*n* = 6 per group), following the experimental approach described by Orilisi et al. [28]. The teeth were obtained from the University of Iowa College of Dentistry and Dental Clinics (Iowa City, IA, USA) after extraction for clinical reasons. Their use for research was conducted in accordance with the local Ethics Committee guidelines and the principles of the Declaration of Helsinki (1964), and written informed consent was obtained from all donors.

After extraction, the teeth were cleaned in an ultrasonic bath with distilled water for 2 min to remove biological debris. Each tooth was examined under magnification to confirm the absence of caries, enamel hypoplasia, cracks, fractures, or other structural defects.

The crowns were sectioned mesiodistally from the occlusal surface using a precision diamond saw (Isomet 1000, Buehler Ltd., Lake Bluff, IL, USA) under continuous water irrigation to separate the buccal and lingual portions. One standardized enamel–dentin specimen was obtained from the central buccal region of each tooth, ensuring that each specimen represented an independent experimental unit. The specimens were prepared with a standardized thickness of 3.0 mm.

The enamel surfaces were sequentially polished using a rotary polishing machine (Arotec, São Paulo, SP, Brazil) with silicon carbide abrasive papers of increasing grit sizes (600, 800, 1200, and 4000). Final polishing was performed using felt discs (3M Brasil, Sumaré, SP, Brazil) and diamond suspensions with particle sizes of 1.0, 0.50, and 0.25 μm (Erios, São Paulo, SP, Brazil) [28,29].

#### 2.3.2. Bleaching Treatment

The enamel specimens were bleached using a 35% hydrogen peroxide gel (Whiteness HP, FGM Produtos Odontológicos, Joinville, SC, Brazil). The gel was prepared according to the manufacturer’s instructions by mixing three drops of hydrogen peroxide with one drop of thickener. A standardized volume (0.06 mL) was applied to the enamel surface using a positive-displacement micropipette designed for viscous liquids (Microman E, Gilson Inc., Middleton, WI, USA).

The exposure time varied according to the experimental group. Each group underwent three bleaching sessions performed at 7-day intervals. Between sessions, the specimens were stored in humidified chambers to maintain moisture.

The experimental groups were defined as previously described.

#### 2.3.3. Surface Microhardness Analysis

Enamel surface microhardness was evaluated using a microhardness tester (HMV-2000, Shimadzu Corporation, Kyoto, Japan) equipped with a Knoop indenter. Indentations were performed under a 25 g static load for 10 s, and Knoop microhardness values (KHN) were calculated using the CAMS-WIN software 2.x (New Age Industries, Southampton, PA, USA). For each specimen, three indentations were performed in the central enamel. To ensure independent measurements and avoid interference between indentations, the specimen was rotated by 120° between successive indentations. The mean Knoop microhardness value of the three indentations was calculated for each specimen. Based on the initial microhardness measurements, specimens with values closest to the overall mean (332.1 KHN) were selected and randomly allocated to the experimental groups to ensure baseline homogeneity [29].

#### 2.3.4. Surface Roughness and Morphology Analysis by 3D Laser Scanning Confocal Microscopy

Prior to the experimental treatments, baseline surface roughness was assessed using a benchtop roughness tester (SJ-401, Mitutoyo, Kawasaki, Japan) to verify the initial standardization of the enamel surfaces and comparability among the experimental groups. The specimens showed comparable baseline Ra values, with an overall mean of approximately 0.03 µm and no statistically significant differences among groups (*p* > 0.05).

After the bleaching procedures, surface characterization was performed using a laser scanning confocal microscope (LSCM; VK-X200 model, Keyence Corporation, Osaka, Japan), which detects height variations based on the intensity of reflected light from the sample surface.

The analyses were performed using a Nikon 150×/0.95 objective lens (OFN25WD 0.2; Nikon Corporation, Minato, Tokyo, Japan). Surface topography was scanned along the *Z*-axis, and the acquired data were processed to reconstruct three-dimensional (3D) images and calculate the roughness parameters Ra (average roughness) and Rz (maximum profile height).

The focal plane was automatically determined based on the highest reflected light intensity. For each specimen, three measurements were obtained from different surface areas, and the mean value was calculated. Representative images from each group were also recorded for qualitative analysis of enamel surface morphology [28].

#### 2.3.5. Energy Dispersive X-Ray Spectroscopy

For the chemical characterization, the specimens (*n* = 6) were mounted on metallic stubs, sputter-coated with gold and analyzed using Energy-Dispersive Spectroscopy (EDS) coupled to a scanning electron microscope (SEM; TESCAN VEGA3, TESCAN, Brno, Czech Republic) equipped with an EDAX microanalysis system.

The analyses were performed at a working distance of 15 mm, accelerating voltage of 25 kV, and magnification of 400×. Phosphorus (P) and calcium (Ca) concentrations were quantified and expressed as weight percentages (wt%) [28].

Calcium (Ca) and phosphorus (P) contents were expressed as weight percentages (wt%) based on EDS measurements. The Ca/P weight ratio was calculated directly from the corresponding Ca and P weight percentages (Ca wt%/P wt%). No conversion to molar or atomic proportions was performed. Accordingly, the Ca/P values were interpreted exclusively as relative indicators of elemental composition and were not compared with the theoretical atomic Ca/P ratio of stoichiometric hydroxyapatite.

### 2.4. Statistical Analysis

After data tabulation, descriptive and exploratory analyses were performed to assess data distribution and homogeneity. Normality and homogeneity of variances were evaluated using the Shapiro–Wilk and Levene tests, respectively.

For Phase I, ATR-FTIR and reactive oxygen species (ROS) data were summarized descriptively as mean and standard deviation. As described above, the five aliquots per experimental condition were technical subsamples derived from a single pooled enamel preparation and therefore did not represent independent biological replicates. Accordingly, no inferential statistical comparisons were performed for Phase I, and these findings were interpreted as exploratory mechanistic observations obtained under standardized substrate conditions.

For Phase II, individual tooth specimens represented independent experimental units (*n* = 6 per group). Surface roughness (Ra and Rz) and energy-dispersive X-ray spectroscopy (EDS) data were analyzed using one-way analysis of variance (ANOVA) to evaluate differences among treatment groups, followed by Tukey’s post hoc test when appropriate. Surface microhardness data were analyzed using two-way repeated-measures ANOVA to evaluate the effects of treatment, evaluation time, and their interaction, followed by Tukey’s post hoc test for multiple comparisons.

A post-hoc power analysis was performed for selected Phase II outcomes considering four experimental groups, six independent specimens per group, and α = 0.05. Based on the observed effect sizes, Cohen’s *f* values were 0.91 for Ra, 1.36 for Rz, and 0.82 for final surface microhardness, corresponding to achieved statistical powers of 94.0%, >99%, and 87.8%, respectively.

All statistical analyses were performed using SigmaPlot software (version 12.0; Systat Software Inc., San Jose, CA, USA). The significance level was set at 5% (α = 0.05).

## 3. Results

Because the five aliquots analyzed per experimental condition in Phase I represent technical replicates derived from a single pooled enamel preparation rather than independent biological replicates, inter-group statistical comparisons were not performed for the Phase I timepoint data (Initial, S1, S2, and S3). Accordingly, the following Phase I results are presented descriptively, consistent with the exploratory nature of this phase as described in Section 2.2.1 and Section 4.

Regarding PO_4_^3−^ peak intensity, the experimental conditions showed similar numerical values at baseline. After the first bleaching session (S1), G45 showed the highest numerical peak intensity, followed by G15, G15SP, and NC. At S2 and S3, G45 continued to show the highest numerical values. The peak intensities observed for G15 and G15SP at these timepoints were also numerically higher than their respective baseline values, while NC showed only small numerical variations across the evaluation periods.

For the PO_4_^3−^ peak area, the experimental conditions showed similar numerical values at baseline. After S1, NC showed the largest numerical peak area, whereas G15SP showed the lowest. At S2, NC continued to show the largest numerical peak area, G15SP showed an intermediate value, and G45 showed the lowest value. A similar numerical ordering was observed after S3, with NC showing the highest peak area, G15 and G15SP showing intermediate values, and G45 showing the lowest value. Across the evaluation periods, the numerical values for NC varied only slightly, whereas lower peak-area values relative to baseline were observed for the bleaching conditions, particularly for G45 (Table 1).

Regarding CO_3_^2−^ peak intensity, similar values were observed among the experimental conditions at baseline. After the first bleaching session (S1), G45 showed the highest peak intensity, whereas G15, G15SP, and NC exhibited lower values. At S2, a progressive pattern was observed across the experimental conditions, following the order G45 > G15 > G15SP > NC. After the third session (S3), G45 maintained the highest peak intensity, followed by G15 and G15SP, whereas NC showed the lowest values. Across the evaluation periods, NC remained relatively stable, while the bleaching conditions showed a progressive increase in CO_3_^2−^ peak intensity, with the most pronounced pattern observed in G45.

For the CO_3_^2−^ peak area, similar values were observed among the experimental conditions at baseline. After S1, the values followed the order NC > G15SP > G45 > G15. At S2, NC showed the largest peak area, G15SP and G15 showed intermediate values of similar magnitude, and G45 exhibited the lowest values. After S3, the observed pattern was NC > G15SP > G15 > G45. Across the evaluation periods, NC remained relatively stable, whereas G45, G15, and G15SP showed a progressive reduction in CO_3_^2−^ peak area, with the lowest post-treatment values observed for G45 (Table 2).

The PO_4_^3−^/CO_3_^2−^ ratio based on peak intensity (Figure 1) showed relatively stable values for NC throughout the experimental period. In contrast, G45 exhibited a progressive reduction across the bleaching sessions and showed the lowest ratio values among the experimental conditions after treatment. G15 also showed a decreasing trend over time, although less pronounced than that observed for G45. G15SP maintained values close to those observed for NC throughout the evaluation periods. Overall, the greatest treatment-associated reduction in the PO_4_^3−^/CO_3_^2−^ peak intensity ratio was observed for G45, whereas G15SP showed the smallest variation relative to baseline.

When the PO_4_^3−^/CO_3_^2−^ ratio was calculated based on peak area, NC again remained relatively stable throughout the experimental period. G45 showed a progressive increase across the bleaching sessions and exhibited the greatest variation relative to baseline. G15 also showed an increasing trend over time, although less pronounced than that observed for G45. In contrast, G15SP maintained values close to those observed for NC, with only minor variation across the evaluation periods (Figure 1).

### 3.1. Analysis of Reactive Oxygen Species (ROS) Production

Figure 2 illustrates the release of reactive oxygen species (ROS) from the bleaching gels, evaluated at the initial time point, immediately after gel manipulation, and at the end of the bleaching treatment through the analysis of the supernatant collected after the reaction. Initially, the G15SP treatment showed the highest ROS production. The G45 and G15 groups presented statistically similar values, both lower than G15SP but higher than the control group (NC).

At the end of the treatment, the G15 group showed the lowest ROS consumption, maintaining a high concentration of these species in the supernatant. The G15SP group, despite having the same application time as G15, exhibited a significantly greater ROS consumption. Finally, the G45 group, which remained in contact with the bleaching gel for a longer period, showed the highest ROS consumption, differing significantly from all other groups (*p* = 0.01).

### 3.2. Surface Roughness and Microhardness Analysis

Table 3 presents the results of surface roughness (Ra and Rz) and surface microhardness (KHN). Regarding the Ra parameter, the G45 and G15 groups showed similar values and exhibited the highest roughness. The G15SP group presented intermediate values, while the control group (NC) showed the lowest Ra values (*p* < 0.05). For the Rz parameter, the G45 group differed significantly from the other groups, followed by G15. The G15SP showed intermediate group values and did not differ statistically from the control group (NC).

Regarding microhardness, at baseline the overall mean of the specimens was 332.1 KHN, with no significant differences among the groups (*p* > 0.05). At the final evaluation, the lowest values were observed in the G45 group, followed by G15 (*p* < 0.05). The G15SP group showed higher values and differed from the other treatments (*p* < 0.05). Over time, only the G45 and G15 groups exhibited a significant reduction in microhardness (Table 3).

### 3.3. Enamel Topographic Analysis

Representative images obtained by 3D laser confocal microscopy allowed the evaluation of enamel surface topography. In the control group (NC), minimal height variation of approximately 0.3 µm was observed, indicating surface stability. The G45 group showed the greatest topographic variation, reaching approximately 2 µm, suggesting more pronounced surface modification. The G15 group exhibited a maximum variation of approximately 0.6 µm, whereas the G15SP group showed values similar to those observed for the control group (≈0.3 µm) (Figure 3).

### 3.4. Energy Dispersive X-Ray Spectroscopy (EDS)

Figure 4 shows the concentrations of phosphorus (P) and calcium (Ca). The results indicate that none of the treatments influenced the *p* values (*p* > 0.05). Regarding Ca, higher values were observed in the control group (NC), which differed significantly from G45 (*p* = 0.02), indicating a reduction in this group. However, no statistically significant difference was observed between G45 and G15 (*p* = 0.06). The G15SP group showed Ca concentrations similar to those of both the control group and the other treatments (*p* = 0.07) (Figure 4).

The Ca/P ratio was calculated to evaluate the inorganic composition of the enamel. The G45 group exhibited a significant reduction compared with the other groups (*p* = 0.01). Although the G15 group also showed a reduction, this difference was not statistically significant (*p* = 0.07). The NC and G15SP groups presented similar Ca/P ratios (*p* = 0.07) (Figure 5).

## 4. Discussion

Although in-office dental bleaching is considered a minimally invasive procedure, the application of gels containing high concentrations of H_2_O_2_ (30–40%) for 30–45 min may induce structural alterations in dental tissues due to the non-selective oxidation of both organic and inorganic components [17]. In this context, the present study evaluated alternative bleaching protocols combining enzymatic catalysts with a 35% H_2_O_2_ bleaching gel to preserve treatment efficacy while minimizing deleterious effects on enamel.

Reductions in enamel microhardness and increases in surface roughness have been associated with bleaching procedures, although the available evidence remains controversial [10,30,31,32], possibly due to methodological differences and variations among bleaching products. To minimize these sources of variability, a multidimensional approach integrating ATR-FTIR, ROS quantification, EDS, surface microhardness, and three-dimensional confocal microscopy was employed, allowing the correlation of chemical, structural, and mechanical alterations in enamel.

Overall, the results suggest that modulation of hydrogen peroxide activity influences the mineral composition and structural behavior of enamel. The exploratory findings from Phase I showed patterns consistent with the structural alterations observed in Phase II, supporting a potential relationship between oxidative activity and structural changes.

ATR-FTIR analysis showed descriptive patterns of progressive changes in the PO_4_^3−^ and CO_3_^2−^ bands in the conditions exposed to H_2_O_2_, particularly G45. These spectral patterns suggest that prolonged exposure to high concentrations of hydrogen peroxide may affect the relative contribution of phosphate- and carbonate-associated bands in enamel. Given the exploratory nature of Phase I, these findings should be interpreted as treatment-associated spectral patterns rather than as inferential evidence of differences among independent biological samples.

The PO_4_^3−^/CO_3_^2−^ ratio showed a similar descriptive pattern, with the greatest variation observed in G45, whereas G15SP remained closer to the untreated control. Previous studies have shown that bleaching agents may modify the relative contribution of phosphate- and carbonate-associated components in enamel [33,34]. Carbonate may substitute either phosphate or hydroxyl groups within hydroxyapatite, whereas phosphate plays an important role in mineral stability and resistance to acid dissolution [35,36,37,38,39,40]. Thus, the spectral patterns observed in the present study may reflect treatment-associated modifications in enamel mineral composition, although these findings should be interpreted within the exploratory nature of the pooled-enamel analysis.

Although slight variations in the intensity of some bands, particularly carbonate, were observed, these changes most likely reflect modifications in the spectral profile rather than a true increase in mineral content. Therefore, the combined analysis of band areas and spectral ratios proved to be a more reliable approach for inferring changes in enamel mineral composition.

EDS analysis further supported these findings by demonstrating reduced calcium content and a lower Ca/P weight ratio in G45, indicating greater alteration of the relative elemental composition of the enamel surface. These changes may reflect mineral loss associated with prolonged exposure to hydrogen peroxide, which can diffuse through the interprismatic spaces of enamel and contribute to demineralization and increased surface porosity [40,41,42]. This interpretation is consistent with the complementary surface and chemical findings observed in the present study, which collectively indicate greater enamel alteration after the conventional 45-min bleaching protocol. Nevertheless, because EDS provides semi-quantitative elemental information rather than direct characterization of crystal structure, changes in Ca content and Ca/P weight ratio should not be interpreted as direct evidence of altered hydroxyapatite crystallinity.

Surface microhardness and roughness analyses corroborated this behavior, with G45 showing a significant increase in roughness and a reduction in microhardness after treatment. These findings indicate that prolonged exposure to hydrogen peroxide promotes degradation of the mineral matrix and increased surface irregularities, ultimately compromising the mechanical integrity of enamel.

In contrast, G15SP exhibited Ca/P ratio, microhardness, and roughness values similar to those of the control group, suggesting greater structural preservation. These findings indicate that the polymeric scaffold may modulate the interaction between hydrogen peroxide and enamel, thereby reducing the mineral and mechanical impact of the bleaching procedure.

When considered together, the FTIR, EDS, and mechanical property data revealed a consistent association between alterations in the mineral phase and structural impairment of enamel. In general, the groups showing the greatest spectroscopic changes also exhibited poorer mechanical and morphological performance.

ROS analysis provided additional mechanistic support for the structural findings. The conventional protocol (G45) exhibited the highest ROS consumption after treatment, suggesting greater intensity of oxidative reactions during bleaching. The G15 protocol showed a smaller variation in ROS levels, possibly related to the shorter exposure time to the bleaching gel.

Interestingly, G15SP exhibited greater initial ROS generation, suggesting that horseradish peroxidase enhanced the catalytic decomposition of hydrogen peroxide, thereby modulating its release kinetics. This behavior indicates that the polymeric scaffold may influence not only peroxide diffusion but also the dynamics of reactive oxygen species generation. Although the G15SP protocol exhibited greater initial ROS generation, this response was not accompanied by increased mineral or structural damage. Instead, the preservation of enamel properties suggests that the enzymatic system promoted a more efficient conversion of hydrogen peroxide into reactive oxygen species during the early stages of the bleaching reaction, thereby reducing prolonged exposure of enamel to residual free H_2_O_2_. In this context, the polymeric scaffold may have acted as a controlled delivery platform, regulating the interaction between HRP and hydrogen peroxide while promoting a more favorable oxidative profile. Consequently, the oxidative potential required for bleaching could be achieved with lower residual peroxide availability, minimizing undesirable effects on mineralized tissue [38,39,40,41,42].

These findings are consistent with the structural results, as G15SP exhibited smaller alterations in ATR-FTIR, EDS, microhardness, and surface roughness. Collectively, the data suggest that modulation of ROS generation and diffusion may reduce the oxidative impact on enamel while preserving its structural integrity without compromising bleaching potential. Furthermore, the comparison between G15 and G15SP indicates that the improved preservation of enamel cannot be attributed solely to the shorter application time. Although reducing the exposure period alone attenuated some structural changes compared with the conventional protocol, the incorporation of HRP within the polymeric scaffold resulted in values consistently closer to those of the untreated control. These findings suggest that enzymatic catalysis contributes beyond time reduction by optimizing hydrogen peroxide decomposition and enhancing bleaching-related reactions while limiting prolonged peroxide interaction with enamel.

Overall, the findings demonstrate that the structural alterations observed after bleaching are closely associated with the chemical and oxidative changes induced by the different protocols, highlighting the importance of modulating hydrogen peroxide reactivity to preserve enamel integrity. Accordingly, all the proposed hypotheses were supported. Compared with the conventional in-office bleaching protocol, the catalytic strategy better preserved enamel mechanical properties by reducing surface roughness and maintaining higher microhardness values. This protective effect was further supported by the smaller chemical alterations detected by ATR-FTIR and the better preservation of enamel elemental composition observed by EDS. Moreover, the greater initial generation of reactive oxygen species in the G15SP group suggests that enzymatic catalysis enhanced hydrogen peroxide reactivity while minimizing its detrimental effects on the dental substrate.

Collectively, these findings provide mechanistic evidence that catalytic modulation of hydrogen peroxide can preserve enamel integrity without compromising the oxidative processes required for bleaching. Thus, strategies based on reducing hydrogen peroxide application time or incorporating catalytic systems may represent promising alternatives to improve the safety of in-office bleaching protocols while maintaining their effectiveness. From a clinical perspective, preserving enamel microhardness, surface roughness, and mineral composition is particularly relevant because these properties are directly associated with the long-term integrity of the tooth surface. Surface alterations induced by bleaching may increase susceptibility to biofilm accumulation, extrinsic staining, erosive challenges, and mechanical wear. Therefore, protocols capable of maintaining these properties while reducing chairside application time may improve the overall safety profile of bleaching treatments without compromising their therapeutic outcome.

The present study simultaneously integrated ROS generation, ATR-FTIR, EDS, surface microhardness, surface roughness, and 3D confocal microscopy to comprehensively evaluate the effects of enzymatically catalyzed in-office bleaching using a polymeric scaffold. By integrating these complementary analytical techniques, it was possible to establish mechanistic correlations between oxidative activity, mineral composition, and structural integrity, providing a more comprehensive understanding of how catalytic modulation of hydrogen peroxide influences enamel preservation than studies based on isolated outcome measures.

The present findings demonstrate the potential of enzymatic catalysis associated with a polymeric scaffold to preserve enamel integrity. Although bleaching efficacy was not directly evaluated in the present study through color-change measurements, previous studies from our research group have demonstrated that this enzymatically catalyzed bleaching strategy provides bleaching effectiveness comparable to the conventional protocol while reducing the application time. Therefore, the potential for maintaining bleaching efficacy discussed herein is supported by these previously published findings rather than by direct color measurements obtained in the present investigation.

Despite these findings, this study has some limitations. The effects of saliva, including its potential demineralizing and remineralizing effects, and the cytotoxicity of the experimental bleaching gels were not evaluated. Future studies should incorporate artificial saliva storage conditions, cytotoxicity assays, and different hydrogen peroxide concentrations and application times to further optimize the safety and clinical performance of enzymatically catalyzed bleaching protocols. Additionally, the use of pooled enamel in Phase I represents a methodological limitation. Although pooling was intentionally adopted to provide a homogeneous enamel substrate across experimental conditions and minimize inter-specimen variability, it precluded the assessment of inter-individual biological variability and resulted in technical rather than independent biological replication. Therefore, the findings from Phase I should be interpreted as exploratory mechanistic evidence obtained under standardized substrate conditions rather than as population-level estimates of treatment effects.

## 5. Conclusions

The results demonstrate that enzymatic catalysis combined with the polymeric scaffold modulates hydrogen peroxide reactivity, resulting in better preservation of enamel mineral composition, surface morphology, and mechanical properties compared with the conventional bleaching protocol. Although higher initial generation of reactive oxygen species (ROS) was observed, this response was associated with reduced structural alterations, indicating a more efficient oxidative process rather than increased enamel damage. While bleaching efficacy was not directly assessed in the present study, previous investigations from our research group have demonstrated that this catalytic strategy achieves bleaching effectiveness comparable to the conventional protocol with a reduced application time. Therefore, the present findings support the potential of this approach to enhance the biological safety of in-office bleaching, although its clinical applicability should be confirmed in future randomized clinical trials.

## Figures and Tables

**Figure 1:**
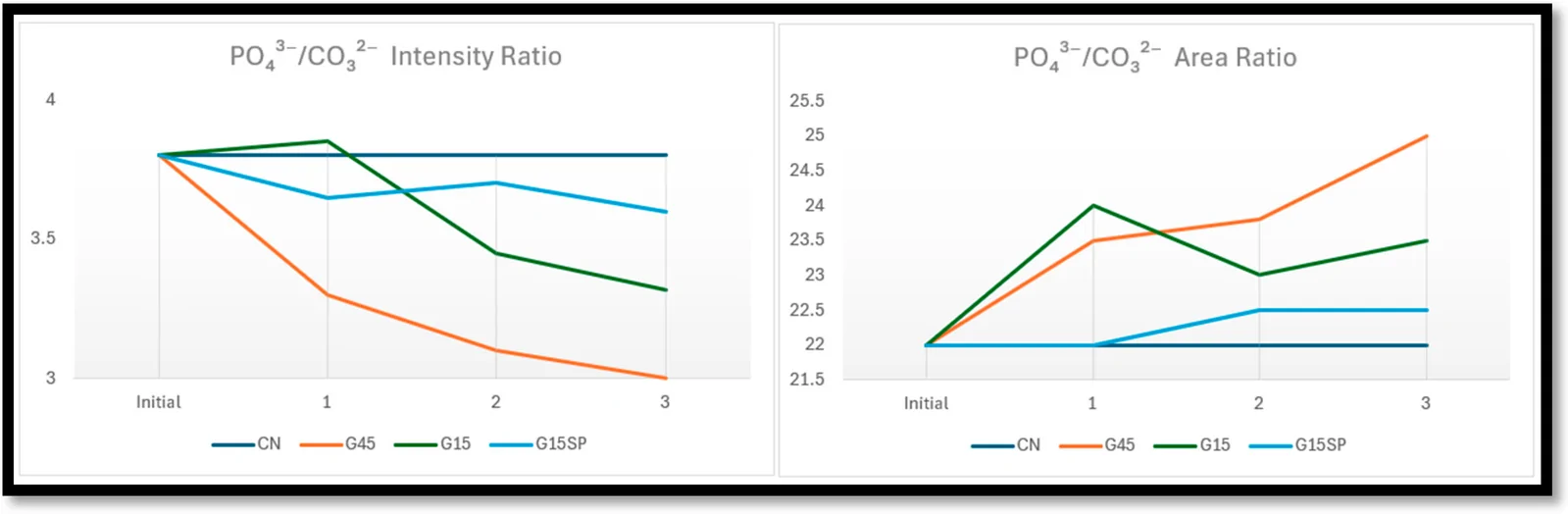
Representation of peak intensity and peak area in the ratio PO_4_^3−^/CO_3_^2−^ *x*-axis: analysis times; *y*-axis: intensity and area values of the PO_4_^3−^/CO_3_^2−^ ratio.

**Figure 2:**
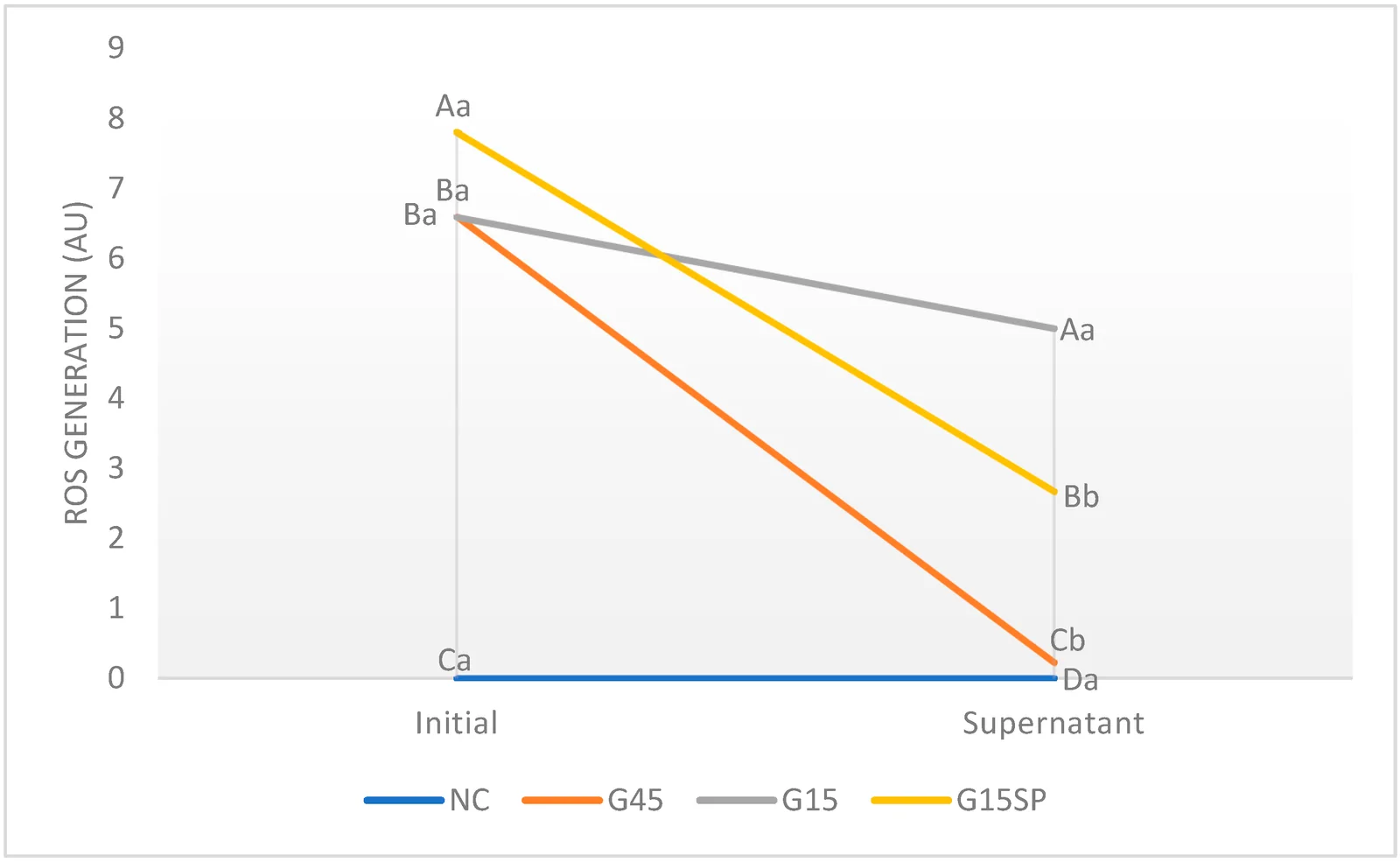
Reactive oxygen species (ROS) generation in the bleaching gel before application (*Initial*) and in the supernatant (Supernatant) for the different experimental protocols. Different uppercase letters indicate significant differences among groups at the same evaluation time, whereas different lowercase letters indicate significant differences between evaluation times within the same group (*p* < 0.05).

**Figure 3:**
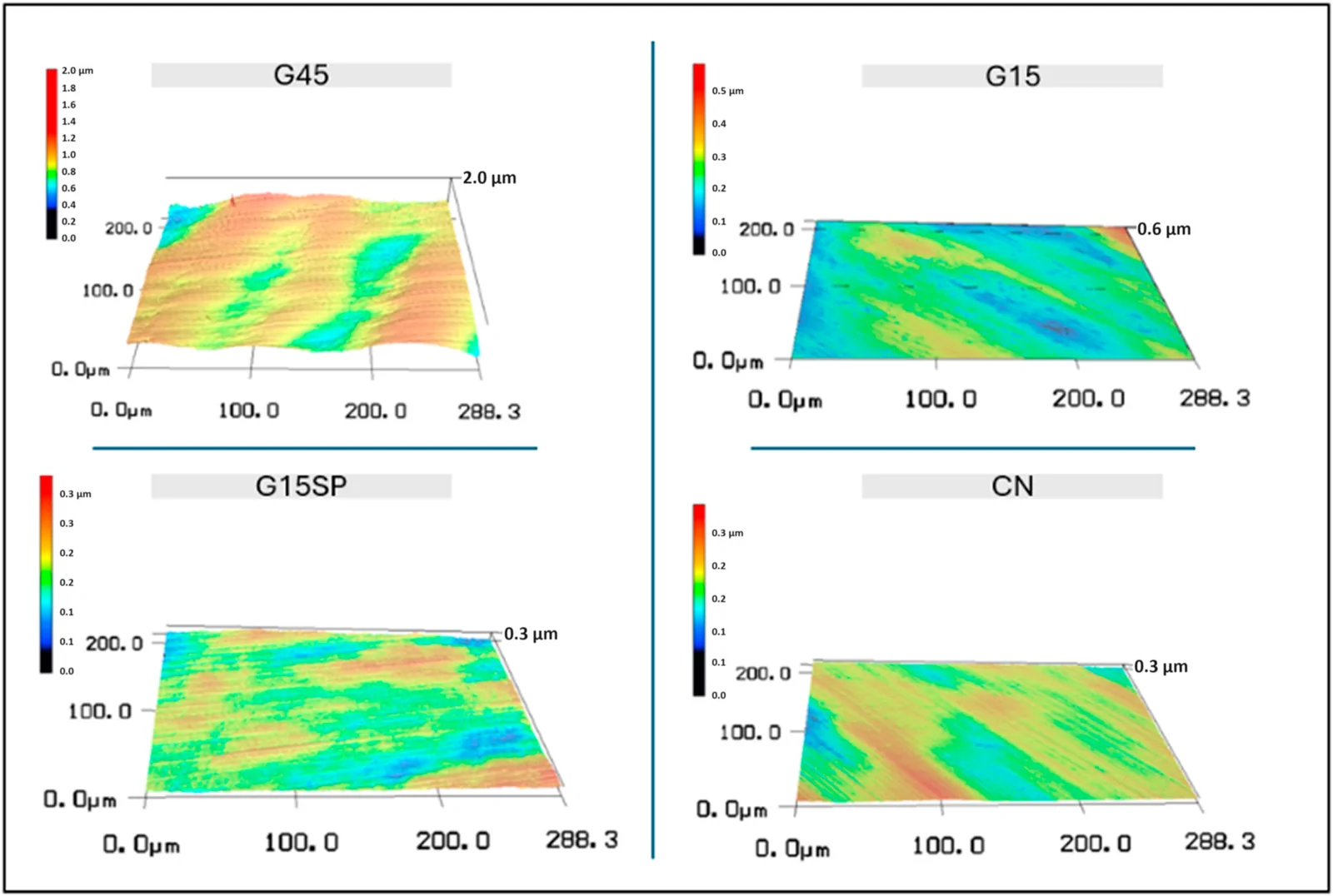
Representative three-dimensional confocal microscopy images of enamel surfaces after the different bleaching protocols. The G45 group showed the greatest surface alterations, whereas G15SP exhibited a surface morphology similar to the negative control (NC).

**Figure 4:**
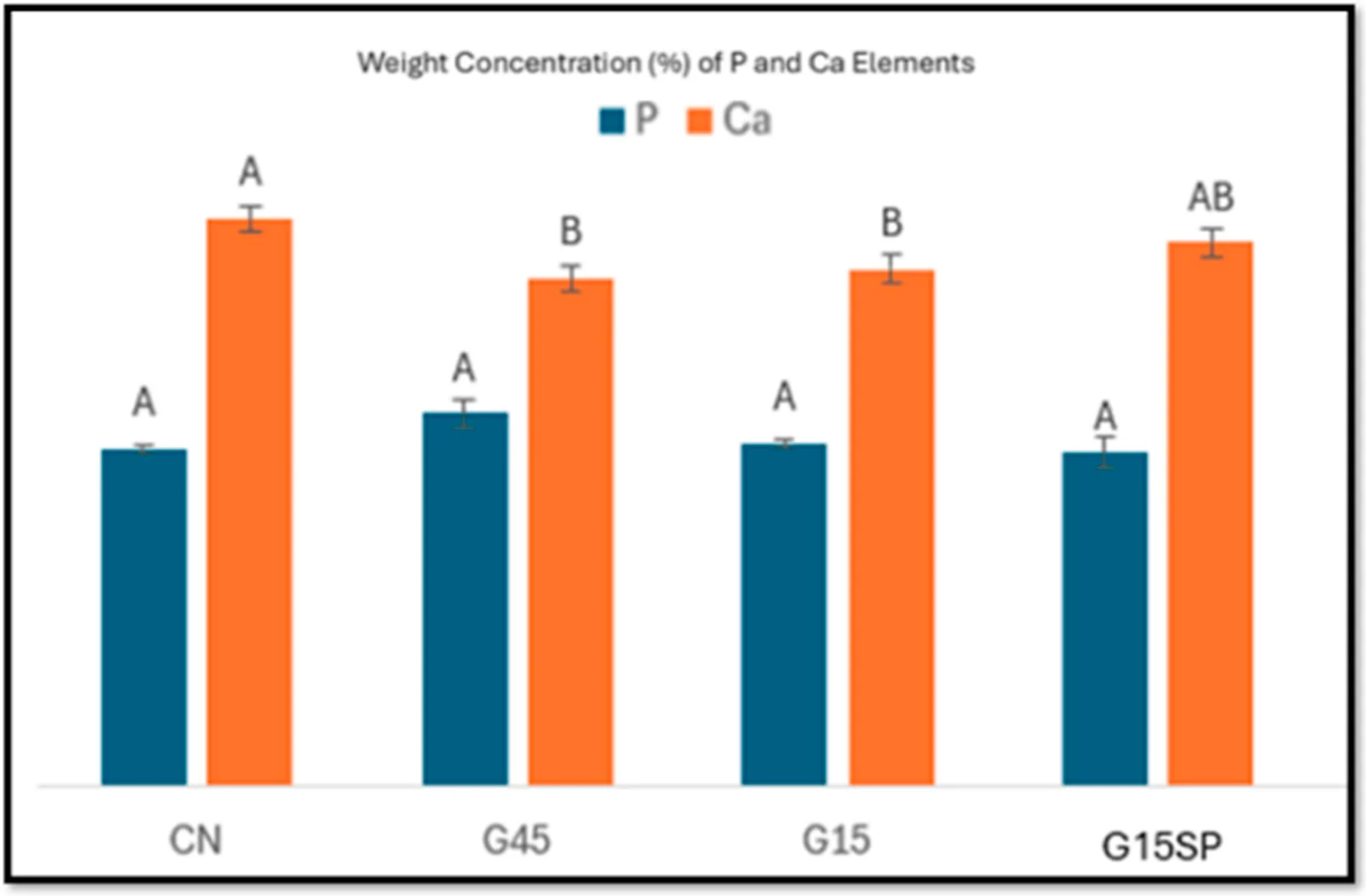
Weight concentration (%) of phosphorus (P) and calcium (Ca) in enamel after the different bleaching protocols. Different uppercase letters indicate significant differences among groups (*p* < 0.05).

**Figure 5:**
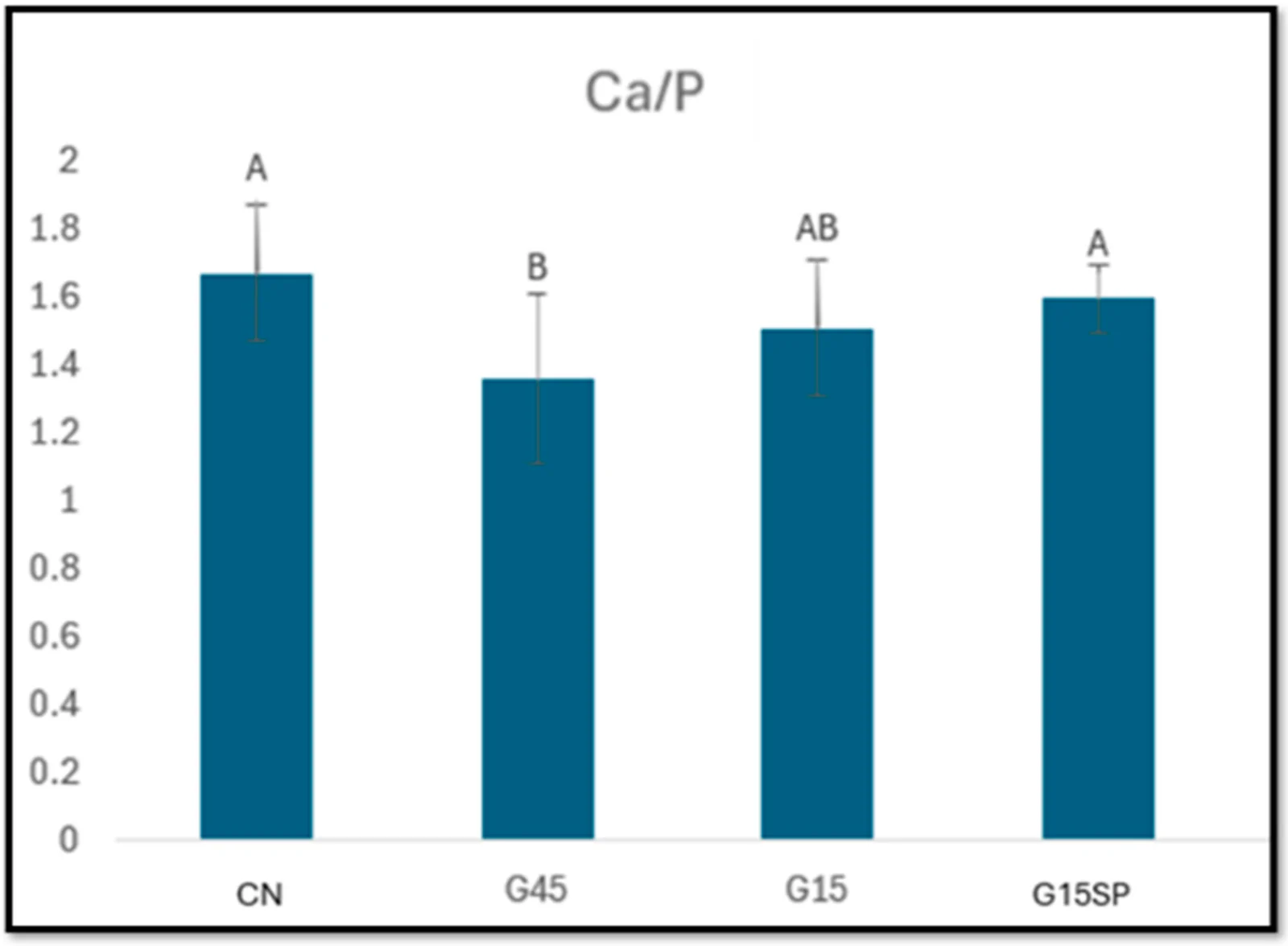
Calcium-to-phosphorus (Ca/P) ratio of enamel after the different bleaching protocols. Different uppercase letters indicate significant differences among groups (*p* < 0.05).

**Table 1:** Mean (±SD) intensity and area of the PO_4_^3−^ peak in enamel according to treatment groups.

**Treatments**	**Intensity**			
	**Initial**	**After 1S**	**After 2S**	**After 3S**
**NC**	0.94 (0.01)	0.94 (0.01)	0.94 (0.01)	0.94 (0.01)
**G45**	0.94 (0.01)	0.96 (0.03)	0.97 (0.02)	0.97 (0.02)
**G15**	0.94 (0.01)	0.96 (0.01)	0.95 (0.01)	0.95 (0.02)
**G15SP**	0.94 (0.01)	0.95 (0.01)	0.95 (0.04)	0.95 (0.04)
**Treatments**	**Area**			
	**Initial**	**After 1S**	**After 2S**	**After 3S**
**NC**	88.81 (0.01)	88.81 (0.01)	88.81 (1.08)	88.81 (0.01)
**G45**	88.81 (0.01)	87.31 (0.85)	84.37 (0.01)	81.17 (2.22)
**G15**	88.81 (0.01)	87.21 (2.32)	85.98 (0.85)	86.26 (1.88)
**G15SP**	88.81 (0.01)	87.20 (1.39)	86.83 (0.50)	86.37 (0.64)

**Table 2:** Mean (±SD) intensity and area of the CO_3_^2−^ peak in enamel according to treatment groups.

**Treatments**	**Intensity**			
	**Initial**	**After 1S**	**After 2S**	**After 3S**
**NC**	0.25(0.01)	0.25(0.01)	0.25(0.01)	0.25(0.01)
**G45**	0.25(0.01)	0.29(0.01)	0.30(0.02)	0.33(0.01)
**G15**	0.25(0.01)	0.25(0.02)	0.28(0.01)	0.29(0.02)
**G15** **SP**	0.25(0.01)	0.26(0.01)	0.26(0.01)	0.27(0.02)
**Treatments**	**Area**			
	**Initial**	**After 1S**	**After 2S**	**After 3S**
**NC**	3.97(0.01)	3.97(0.01)	3.97(0.01)	3.97(0.01)
**G45**	3.97(0.01)	3.75(1.04)	3.57(0.94)	3.19(0.51)
**G15**	3.97(0.01)	3.60(0.40)	3.69(0.25)	3.63(0.24)
**G15** **SP**	3.97(0.01)	3.94(0.04)	3.83(0.05)	3.81(0.13)

**Table 3:** Post-treatment enamel surface roughness (Ra and Rz, µm) according to the experimental groups.

Treatments	Roughness		Microhardness	
	Ra (µm)	Rz (µm)	Initial (KHN)	Final (KHN)
**NC**	0.03 (0.01) ^C^	0.67 (0.15) ^C^	333.72 (15.3) ^Aa^	333.61 (13.2) ^Aa^
**G45**	0.07 (0.03) ^A^	1.12 (0.16) ^A^	332.58 (15.4) ^Aa^	306.32 (18.4) ^Db^
**G15**	0.06 (0.01) ^A^	0.91 (0.13) ^B^	331.42 (12.6) ^Aa^	315.92 (12.2) ^Cb^
**G15SP**	0.04 (0.01) ^B^	0.70 (0.15) ^C^	331.26 (8.1) ^Aa^	329.91 (7.03) ^Ba^

Uppercase letters compare groups and lowercase letters compare timepoints. (One-way ANOVA with Tukey’s post hoc test, *p* < 0.05—Roughness; and two-way repeated measures ANOVA with Tukey’s post hoc test, *p* < 0.05—Microhardness).

## Data Availability

The original contributions presented in this study are included in the article. Further inquiries can be directed to the corresponding author.
